# Observation of a two-dimensional liquid of Fröhlich polarons at the bare SrTiO_3_ surface

**DOI:** 10.1038/ncomms9585

**Published:** 2015-10-22

**Authors:** Chaoyu Chen, José Avila, Emmanouil Frantzeskakis, Anna Levy, Maria C. Asensio

**Affiliations:** 1 Synchrotron SOLEIL, Beamline ANTARES, L'Orme des Merisiers, Saint Aubin-BP 48, Gif surYvette 91192, France

## Abstract

The polaron is a quasi-particle formed by a conduction electron (or hole) together with its self-induced polarization in a polar semiconductor or an ionic crystal. Among various polarizable examples of complex oxides, strontium titanate (SrTiO_3_) is one of the most studied. Here we examine the carrier type and the interplay of inner degrees of freedom (for example, charge, lattice, orbital) in SrTiO_3_. We report the experimental observation of Fröhlich polarons, or large polarons, at the bare SrTiO_3_ surface prepared by vacuum annealing. Systematic analyses of angle-resolved photoemission spectroscopy and X-ray absorption spectra show that these Fröhlich polarons are two-dimensional and only exist with inversion symmetry breaking by two-dimensional oxygen vacancies. Our discovery provides a rare solvable field theoretical model, and suggests the relevance of large (bi)polarons for superconductivity in perovskite oxides, as well as in high-temperature superconductors.

## Abstract (web summary)

A polaron is a quasiparticle formed through the strong interaction between an electron and the ions in a crystalline solid. Here, the authors observe Fröhlich polarons, formed by the coupling of electrons and long-wavelength optical phonons, in strontium titanate.

## Graphical abstract

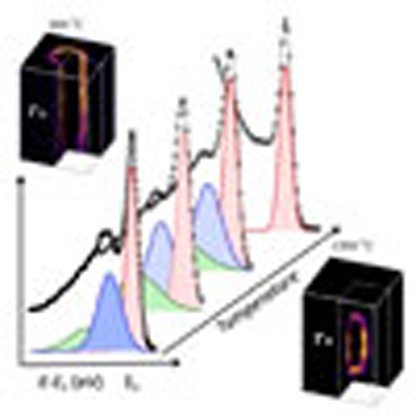

The concept of a polaron was set forth by Landau^1^ in 1933 and has attracted much attention over the following decades. It not only describes the specific physical properties of charge carriers in polarizable solid, but also constitutes an interesting field theoretical model consisting of a fermion interacting with a scalar boson field^2^. Even today, there is ongoing research on polarons, including the basic theory^3^, as well as their behaviour in more compelling situations such as high-temperature superconductors^4^,^5^,^6^ and colossal magnetoresistance in rare-earth manganites^7^.

There are different types of polaron states in solids, such as Fröhlich polaron, small polaron, spin polaron, bipolaron and hydrated polaron^2^. Originally polaron describes interaction between charge carrier and long-wavelength optical phonons, namely, Fröhlich polaron^8^, of which the spatial extension exceeds the lattice constant. Fröhlich polarons with low kinetic energy propagate through the lattice as free electrons but with an enhanced effective mass, thus directly measurable by spectroscopies like angle-resolved photoemission spectroscopy (ARPES).

SrTiO_3_ is the key material in the emerging field of oxide electronics^9^. Structurally or electronically modified SrTiO_3_ presents a wide spectrum of phenomena such as superconductivity^10^, two-dimensional (2D) electron gas (2DEG)^11^,^12^, ferroelectricity^13^ and blue luminescence^14^, indicating versatile interplay of charge, spin, orbital and lattice degree of freedom^15^. However, previous studies give controversial information about the carrier type in SrTiO_3_ (see, for example, refs 16, 17 and references therein), hampering the thorough distinction of these internal interplay. Recently, ARPES study on FeSe film grown on SrTiO_3_ has revealed the enhancement of superconducting transition temperature *T*_c_ due to the coupling between FeSe electron and oxygen optical phonon in SrTiO_3_ (ref. 6).

In this article, by examining the surface electronic structure of vacuum-annealed SrTiO_3_, we report the observation of 2D liquid of Fröhlich polaron, a new quasi-particle formed by conduction band electron coupled with one polar longitudinal optical (LO) phonon mode. By controlling the annealing temperature, we can precisely tune the surface oxygen vacancy concentration, which develops from 2D to 3D distribution of charges. Moreover, by compiling partial electron yield (PEY) and total fluorescence yield (TFY) XAS spectrum, we are able to identify unambiguously these two distinctive distributions of oxygen vacancies. In the 2D case, oxygen defect dipoles break the inversion symmetry and initiate the coupling between electrons and polar LO phonons. This intermediate coupling leads to the formation of a new type of quasi-particles, Fröhlich polarons, whose spectra function contains multiple replicas of original bands, equally spaced *ω*_LO_ apart, with *ω*_LO_ the effective energy of LO phonons, as directly observed by ARPES here. Photon-energy-dependent ARPES measurements reveal the 2D nature of Fröhlich polarons. In the 3D oxygen vacancy case, the 2D polarons collapse into 3D electron liquid due to the reversal of symmetry. Our findings reveal the essential role of surface/interface LO-phonon-related large (bi)polarons in understanding superconductivity in perovskite oxides, as well as in high-temperature superconductors.

## Results

### Tunable oxygen vacancy concentration via vacuum annealing

**Figure 1:**
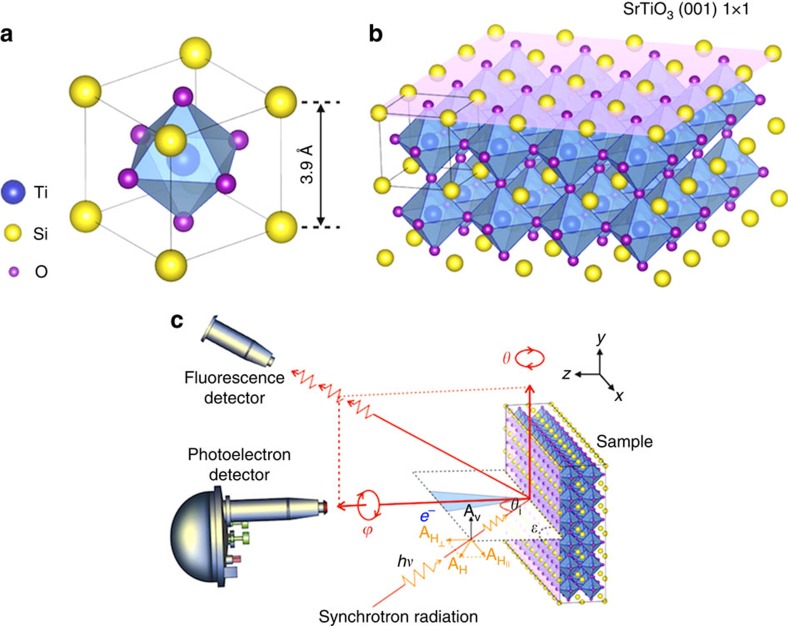
Lattice structure of SrTiO_3_ and measurement geometry. (**a**) The cubic unit cell, with the TiO_6_ octahedron shown as magenta surfaces. O atoms at O1 cites have Ti–O bonds along *z* direction, while O2 cites have Ti–O bonds parallel to the *x–y* plane. (**b**) A 4 × 4 × 2 block of unit cells. The top layer shown in light purple indicates the (001) surface. (**c**) Experimental geometry for XAS and ARPES measurements. **A**_H_(**A**_V_) is the vector potential of the incident light with linear horizontal (vertical) polarization; *θ* and *φ* denote the polar and azimuthal degree of freedom and *θ*_i_ is the polar angle of incidence. The analyser slits permit photoelectron detection along the *x* axis. For all different *θ*, the incident photon momentum, the photoelectron momentum and the surface normal lie on the same plane (*ɛ*). For all the ARPES data, we set the *θ*_i_∼75°, very close to the grazing angle, to enhance the surface contribution. We use linear horizontal polarization (LH). The beam polarization is even to the scattering plane. According to the matrix element effect (MEE), only orbitals with even symmetry respect to the scattering plane can be excited and detected.

Intrinsic SrTiO_3_ is a band insulator with a ∼3.2 eV gap, and experiences a second-order phase transition from cubic to tetragonal structure at the critical temperature of about 105 K (ref. 18). For the present study, both the XAS and ARPES measurements were performed ∼170 K while the low-energy-electron-diffraction (LEED) patterns were taken at room temperature. Thus for our study, the bulk SrTiO_3_ is in the centre-symmetric equilibrium structure, that is, the Ti atom lying in the centre of the regular octahedron of oxygen atoms in the cubic perovskite structure (Fig. 1a). All the experiment data is from the (001) surface as shown in Fig. 1b.

**Figure 2:**
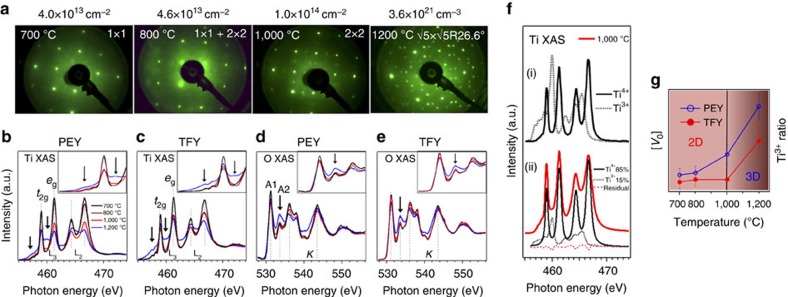
Creating 2D and 3D oxygen vacancies. (**a**) LEED patterns with surface reconstruction labelled for SrTiO_3_ surfaces prepared at different annealing temperature sequentially. The corresponding carrier densities are calculated by Ti^3+^ ratio from PEY XAS spectra for 2D case (≤1,000 °C) and TFY-XAS spectra for 3D case (1,200 °C). See Methods for details. (**b**–**e**) Ti *L*_2,3_-edge and O *K*-edge XAS spectra from different surfaces. Black arrows indicate the peak intensity coming from Ti^3+^. (**f**) Top, numerically calculated XAS spectra of Ti^3+^ and Ti^4+^ ions in SrTiO_3_. Bottom, decomposition of experimental XAS spectra, exemplified by the PEY spectra for surfaces annealed at 1,000 °C. (**g**) Schematic evolution of oxygen vacancy concentration and Ti^3+^ ratio from PEY and TFY spectra. Vertical black line indicates the boundary between 2D and 3D oxygen vacancies.

To start with, we present the precisely regulation of surface carriers by controlling the amount of oxygen vacancies via vacuum annealing. We prove the existence of oxygen vacancies by reproducing the well-known LEED patterns (Fig. 2a and also see, for example, ref. 19 and references in), which reflect the surface reconstruction due to the alignment of oxygen vacancies. Charge transfer from oxygen vacancies to Ti atoms (consequently, Ti^4+^ to Ti^3+^) allow us to derive the oxygen vacancy concentration [*V*_O_ ] from the XAS spectra of Ti *L*_2,3_ edge, attributed to excitations of 2*P*_3/2_ and 2*P*_1/2_ subshells to unoccupied *t*_2g_ and *e*_g_ states, as shown in Fig. 2b,c. As indicated by the black arrow and more clearly shown in the insets, a 460 eV peak gradually increases with annealing temperature. According to the theoretical simulation^20^ (Fig. 2f, top), this peak comes from the Ti^3+^ *L*_3_ edge. Since X-ray absorption near-edge spectroscopy is an atomic probe, strongly sensitive to its formal oxidation state and coordination chemistry^21^, we can calculate the Ti^3+^/Ti^4+^ ratio by assuming the linear combination of Ti^4+^ and Ti^3+^ signals under the single inelastic electron scattering condition^22^,^23^. As exemplified in Fig. 2f (bottom) for the PEY XAS spectra from surface annealed at 1,000 °C, the experimental spectra can be decomposed into 85% Ti^4+^ and 15% Ti^3+^ , using multiple least-squares fit. Figure 2g schematically summarizes the fitted PEY ratio corresponding to surfaces with different annealing temperature. The monotonic increase of oxygen vacancies with annealing temperature clearly demonstrates that we can control and reproduce the carrier concentration of the SrTiO_3_ samples. This point has been further corroborated by the agreement between the Ti^3+^ /Ti^4+^ ratio calculated by PEY XAS and Ti 3p core levels shown in the Supplementary Fig. 1. Notice that we have chosen a similar energy window (∼40 eV) to collect secondary electrons for XAS spectra and core level photoelectrons spectra, ensuring similar depth sensitivity in both data sets.

In addition, we can also prove the increase of surface oxygen vacancies from the O *K*-edge XAS spectra (Fig. 2d,e). As shown clearly in the insets, the A2 peak increases dramatically for the surface annealed at 1,200 °C. This peak arises from the Ti–O *pd-*hybridization with a *σ*-bonding character. Its increase indicates the lattice distortion caused by oxygen vacancies, in qualitatively agreement with our conclusion based on Ti *L*_2,3_-edge data. Given that each oxygen vacancy nominally transfers two electrons to the Ti *d* band^24^, and there are three O atoms but only one Ti atom in the unit cell, Ti^3+^ ratio is six times the value of the oxygen vacancy concentration [*V*_O_ ] (Fig. 2g), which allows XAS on Ti *L*_2,3_ edge be much more sensitive than O *K* edge. Thus it is sensible to calculate the [*V*_O_ ] from the XAS spectra of Ti *L*_2,3_ edge.

More importantly, we show that the oxygen vacancy distribution displays a clear dimensionality change. Figure 2g also summarizes [*V*_O_] values fitted from TFY-XAS spectra. For a given annealing temperature, both PEY and TFY spectra are recorded simultaneously from the same sample. Contrary to the monotonic increase of PEY [*V*_O_ ], the TFY concentration remains at noise level at the beginning and only increases with annealing temperature above 1,000 °C. This distinctive behaviour is based on the different depth sensitivity of PEY and TFY spectra^25^. The PEY signals, that is, energy-selected secondary electrons in present case, typically from an average depth of <1 nm (approximately two unit cells deep), are highly surface sensitive. In contrast, the TFY signals mainly give bulk information due to the long mean free path (MFP) of photons, (∼100 nm). Thus, we conclude that vacuum annealing from 800 °C to 1,000 °C generates 2D oxygen vacancies confined at the surface. However, for samples annealed above 1,000 °C, the high concentration of oxygen vacancies presents a 3D distribution of charges. For the whole annealing temperature range, the concentration of induced surface charge carriers (2D and 3D) increases monotonously with temperature. In addition, judged from the sharp LEED pattern, the vacancies are well-aligned. These ordered defect dipoles break the local inversion symmetry, allow the Raman measurement of first-order phonons^26^ and suggest a phase with ferroelectric-like polarization at the reduced surface of SrTiO_3_ (ref. 27).

### Observation of Fröhlich polaron spectra by ARPES

**Figure 3:**
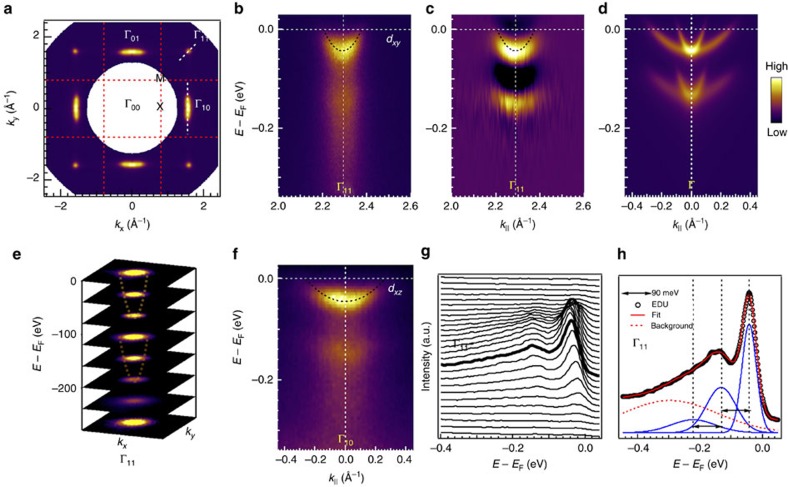
Observation of Fröhlich polaron spectra by ARPES at SrTiO_3_ surface prepared at 700 °C. (**a**) Symmetrized Fermi surface of SrTiO_3_ in *k*_*x*_*−k*_*y*_ space. Γ_10_=2*π/*a(1, 0)∼(1.61, 0) Å^−1^. (**b**) Dispersion from Γ_11_ along [11] direction with *d*_*xy*_ orbital character. Dispersion from Γ_10_ along [01] direction with *d_xz_* orbital character is shown in **f**. The momentum cuts are shown in dashed while lines. (**c**) Energy-second-derivative image of spectra in **b**. (**d**) Simulated large polaron dispersions with *d*_*xy*_/*d*_*yz*_ and *d*_*xz*_ orbital characters. (**e**) Constant-energy contours of large polaron electronic structure, characterized by two (or more) similar electron pockets, indicated by dashed lines. (**g**) EDC stacks of spectra in **b**. Black empty circles indicate the EDC shown in **h**. (**h**) Franck–Condon line shape fitting. The fitted EDC (red line) is composed of three Gaussian peaks (blue line), separated in energy by 90 meV, and a hump-like background (red dashed line). Black dashed lines in **b**,**c** and **f** are fitted dispersions for the zero-order bands. See Supplementary Fig. 2 for details.

It is natural to ask what kind of electronic structure does this ferroelectric-like layer correspond to. Figure 3a shows the symmetrized Fermi surfaces (FSs) from the SrTiO_3_ surface annealed at 700 °C. Thanks to the matrix element effect, we can selectively excite electrons with different orbital symmetry (see Fig. 1c for measurement geometry). No ARPES spectral intensity can be detected at the first Brillouin zone centre (Γ and Γ_00_ specifically). FSs at Γ_10_ and Γ_01_ are elliptical, with *d*_*xz*_ (Fig. 3f) and *d*_*yz*_ orbital character, respectively. The circular FS at Γ_11_ are from *d*_*xy*_ orbital (Fig. 3b). Fitted dispersions (black dashed lines in Fig. 3b,c,f) show an effective mass of ∼0.6*m*_e_ (*m*_e_ the free electron mass) for *d*_*xy*_ band, and ∼6*m*_e_ for *d*_*xz*_ band (see Supplementary Fig. 2 for fitting details). These lighter effective masses will contribute to the high carrier mobility^12^,^28^.

An extraordinary distinction is that there exist replica bands for each orbital (Fig. 3b,f), lying ∼90 meV higher than the original, which can be clearly distinguished from the energy-second-derivative spectra in Fig. 3c. The replica forms one more higher-lying electron pocket, as shown by the constant-energy contours in Fig. 3e. These replicas are attributed to the many-body effect, specifically, the electron–phonon coupling (EPC)^6^,^29^. The characteristic energy, ∼90 meV, corresponds to one surface polar LO phonon mode^30^. These dispersions hence describe electrons weakly coupled to a lattice vibrational mode by the Coulomb interaction, which together can move coherently through the solid and composite a new type of quasi-particle, that is, large polaron. We use the weak-coupling field-theoretical Fröhlich Hamiltonian^8^ as the first approximation to describe EPC in both 3D and 2D cases:


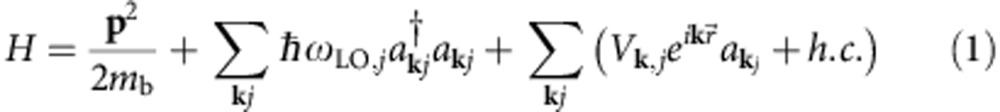


Where **r** is the position coordinate operator of the electron with band mass *m*_b_, **p** is its canonically conjugate momentum operator; *a*_**k***j*_^†^(*a*_**k***j*_) is the creation (annihilation) operator for the *j-*th LO phonons of wave vector **k** and energy *ℏω*_LO,*j*_. The *V*_**k***j*_ is the Fourier component of the EP interaction. Considering one branch of dispersion-less LO-phonon (Einstein model)^31^,^32^,^33^, this interaction system becomes one of the exactly solvable models in many-body physics^34^. The spectra can be well-described by a Franck–Condon model with Poisson distribution:


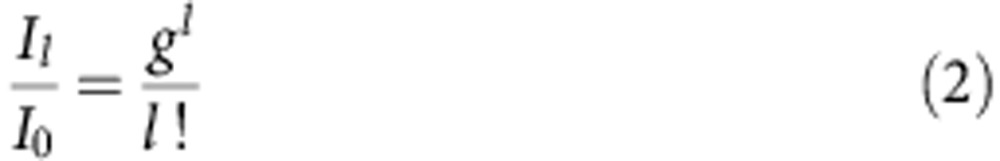


Here *I*_0_ is the quasi-particle peak intensity and *I*_*l*_ the *l-*th replica intensity with *l* phonon(s) dressed. *g* is a constant, related with the EP interaction in 3D.

Considering the ARPES spectra in Fig. 3b,f, the lower-lying band corresponds to charge carriers without dressing phonons, while the higher one corresponds to charge carriers dressing one phonon. Other higher-order bands are nearly invisible but can be deduced from the energy distribution curves (EDCs) fitted with Franck–Condon line shape as shown in Fig. 3h (refs 6, 29). The fitting gives an energy separation *ω*_LO_∼90 meV and *g*=0.85. We can also use other fitting parameters *α*_3D_ and *α*_2D_, to characterize the Fröhlich coupling constant. Inferred from the zero-phonon peak intensity fraction of total weight, according to the diagrammatic quantum Monte Carlo simulation^35^, we have *α*_3D_∼2.5. Using the scaling relation *Z*_2D_ (*α*)=*Z*_3D_ (3*πα/*4) for 2D polarons^36^, we have *α*_2D_∼1.1. Notably, here we use a common and available approach to estimate the 2D coupling strength indirectly. However, although beyond the scheme of present work, a more accurate model directly describing 2D Fröhlich polaron spectra is highly desired.

It is worthy to note that the whole spectra with multiple bands we observed here describe the ground state of Fröhlich polaron, not the excited state, since the temperature is set to zero for the theoretical spectra function^34^. In the ground state of Fröhlich polaron, some probability exists that the charge carriers have different set of energy *ω*_*l*_=*ɛ*_*b*_*-*Δ+*ω*_LO_*l*. Furthermore, this is different from the characteristic ‘peak-dip-hump' spectra in cuprates^37^,^38^, in which case localized small polarons show no high-order dispersion. Our discovery only becomes possible because of the intermediate coupling strength. The discrete phonon mode energy is bigger than the electron bandwidth and most crucially, because the forward scattering is dominating the EPC, as discussed in the recent literature. Figure 3d shows the simulated dispersion of Fröhlich polaron for both *d*_*xy*_ and *d*_*xz/yz*_ orbitals, with the full width at half maximum (FWHM) set as constant, 25 meV and without multiple phonon contribution. Our discovery represents a ‘rare' field theoretical model of Fröhlich polaron, which is exactly solvable^29^,^34^.

One may wonder if these multiple bands arise from band bending effect due to the quantum confinement of the conduction band bottom. In fact, the 2D electron gases/liquids at SrTiO_3_ surface have become model systems for engineering emergent behaviour and studying the quasi-particle dynamics in complex transition metal oxides^11^,^12^,^15^. Here we rule out this possibility due to the fact that the sub-bands in the quantum confinement picture are real electronic structure, crossing Fermi level, but our observed shadow bands are the ‘echo' of EPC, without Fermi level crossing. See Supplementary Fig. 3 for schematic understanding. We also clarify that, contrary to previous works^12^,^39^, during our experiments, all the surfaces displayed no detectable dependence on synchrotron irradiation, as demonstrated by the core level and XAS spectra in Supplementary Fig. 4.

Many seminal studies have been recently done describing the 2DEG scenario, which provide a lot of information even if many important aspects of this phenomenon are still under inflamed discussion. What is important, however, is that there is a quite robust consensus on the conditions in which the 2DEG is stable. Consequently, we have been able to identify three important parameters: carrier density, oxygen vacancy concentration and thickness of the surface layer where the 2DEG takes place. The reported value for the carrier density is close to 10^14^ cm^−2^ (see, for example, refs 11, 15). For the oxygen vacancies, most of the works report a rather constant concentration^11^,^12^,^40^. Finally, the thickness is estimated in most of the important works around four to eight cell units^11^,^12^,^15^,^40^. For the ‘polaron' scenario, however, the formation appears when the carrier density, the oxygen vacancies and the thickness of the surface layer are in rather different ranges. In our manuscript, we have demonstrated that the polarons only form when the carrier density is close to 10^13^ cm^−2^, 1 order of magnitude lower than that for the case of 2DEG. For the oxygen concentration, the polarons appear for approximately half the concentration of oxygen vacancies reported for 2DEG systems (Supplementary Fig. 1). Finally, for the thickness, thanks to the parallel detection of XAS and ARPES we can confirm that the thickness of the surface polarons is not larger than one to two cell units, at least four to eight times smaller than that for the 2DEG surface layer.

### Dimensionality and stability of the Fröhlich polarons

**Figure 4:**
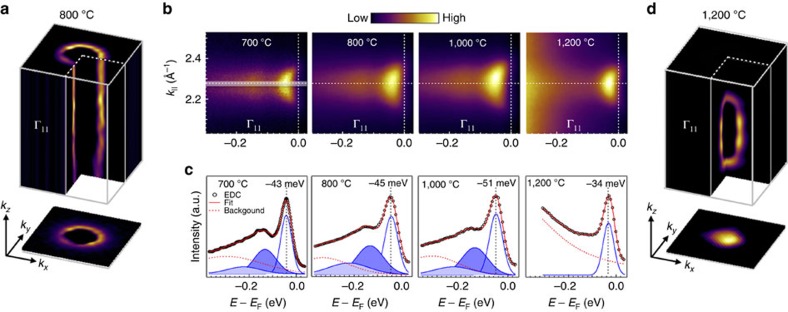
2D nature and stability of large polarons in SrTiO_3_. (**a,d)** Fermi surface of SrTiO_3_ in *k*_*x*_−*k*_*y*_−*k*_*z*_ space with *d*_*xy*_ orbital character. The FSs are centred at one Brillouin zone centre, Γ, which is one high-symmetric point in three-dimensional momentum space with (*k*_*x*_*, k*_*y*_ *, k*_*z*_)=2*π/*a(1, 1, 3)∼(1.61, 1.61, 4.83) Å^−1^. (**b**) Evolution of dispersions from the Γ_11_ cut as in Fig. 3b. (**c**) EDC curves (black empty circles) extracted from the momentum window shown in grey in **b**. Franck–Condon line shape fitted curves are shown with red lines. Blue lines represent individual Gaussian peaks.

As deduced from the XAS spectra, annealing below 1000 °C creates 2D charge carrier localized mainly at the surface. From ARPES spectra we identify these charge carriers as Fröhlich polarons. We then directly demonstrate the 2D nature of Fröhlich polarons by ARPES. Photon-energy-dependent ARPES can map the FS at 3D momentum space (see Methods). Figure 4a shows the FS of *d*_*xy*_ orbital around one bulk Γ point in *k*_*x*_*−k*_*y*_*−k*_*z*_ momentum space from surface annealed at 800 °C. To highlight the detail, we plot the second-derivative photoemission intensity versus momentum. Its cylindrical shape and the lack of dispersion along *k*_*z*_ direction directly confirm the 2D nature of the FS. The projection of this 2D FS onto *k*_*x*_*−k*_*y*_ momentum space composes an open circle. On the contrary, for surface annealed at 1,200 °C, the corresponding *k*_*z*_ dispersion of the FS shows a ellipsoid-like shape, whose projection shows filled circle feature (Fig. 4d), composing evidence of 3D carriers. Consequently, the EDCs analysis as a function of the annealing temperature (Fig. 4c) shows the polaronic bands till the oxygen vacancies present a 3D distribution. In fact, only one Gaussian peak could be fitted to the 1,200 °C EDC, with a non-vanishing background connecting the oxygen vacancy band (Supplementary Fig. 1c). This higher binding energy spectral weight commonly results from electron interaction, reminiscent of liquid behaviour^12^. Thus, both FSs and EDCs spectral analysis prove the 2D nature of Fröhlich polarons. The transition from 2D Fröhlich polaron to 3D electron liquid will be discussed in detail later.

**Figure 5:**
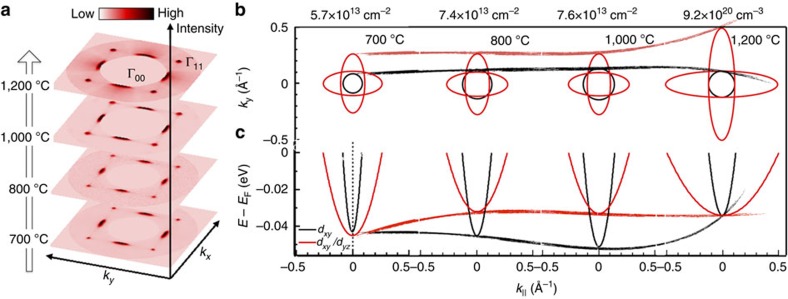
Evolution of Fermi surfaces and dispersions. (**a**) Symmetrized Fermi surfaces of SrTiO_3_ in *k*_*x*_*–* *k*_*y*_ space for (001) surfaces annealed at different temperature. The raw data is shown in Supplementary Fig. 5. (**b**) Fitted FSs for all the three orbitals. The calculated carrier densities are shown for corresponding FSs. See Methods for calculation formula. The superimposed red and black curves indicate the size change for *d*_*xz*_ FS and *d*_*xy*_ FS, respectively. (**c**) Fitted dispersions for different orbitals. See Supplementary Fig. 2 for fitting details. The superimposed red and black curves indicate the band bottom change for *d*_*xz*_ band and *d*_*xy*_ band (zero order), respectively.

Despite its ultimate collapse, 2D Fröhlich polarons are relatively stable over a wide carrier density. The carrier density can be calculated from the Luttinger area of the FSs (see Methods), shown in Fig. 5a,b (see Supplementary Figs 5 and 2 for detailed FSs and fitting). For 2D Fröhlich polaron, we have *n*_2D_∼5.7 × 10^13^ cm^−2^, 7.4 × 10^13^ cm^−2^ and 7.6 × 10^13^ cm^−2^ for surfaces prepared at 700 °C, 800 °C and 1,000 °C, respectively. We have also estimated the carrier density according to the Ti^3+^ /Ti^4+^ ratio from PEY XAS spectra (see Methods). For 2D case, we got *n*_2D_∼4.0 × 10^13^ , 4.6 × 10^13^ and 1.0 × 10^14^ cm^−2^ for corresponding surfaces, in qualitative agreement with ARPES data. It is worthy to note that, for both ARPES and XAS, we assume an ideal 2D scenario. In reality the probe depth for our ARPES and XAS measurements are different since the photoelectron kinetic energy is about 95 eV for ARPES and 40 eV for XAS. As shown in Fig. 4c, from all samples annealed between 800 °C and 1,000 °C, Franck–Condon line shape fitting gave similar second and third order polaronic peak weight, indicating a constant EPC and relatively stable polarons, regardless of the vacancies organization or charge concentration.

Estimated from the 2D carrier density for 700 °C case, the Wigner–Seitz radius *r*_s_=(2*πn*_2D_)^−1/2^ is ∼5.3 Å and the average separation between two Fröhlich polarons is ∼11 Å (approximately three unit cells). Consequently, the momentum width Δ*k* from ARPES spectra at Fermi momentum gives a polaron MFP *λ*∼10 Å. The Franck–Condon fitting in Fig. 4c also gave similar hump-like background for all the three surfaces. Note that, unlike the 1,200 °C case with oxygen vacancy band observed at higher binding energy, this background can only originate from the conduction band, and most likely, attributed to the interaction between polarons. In addition, the effective mass for Fröhlich polaron (∼0.6*m*_e_ for *d*_*xy*_ orbital) is even lighter than that both for 3D liquid in our case (∼1.1*m*_e_) and for 2D electron gas from reported data (∼0.7*m*_e_)^11^, indicating its high mobility. Thus it is proper to conclude that 2D liquid of Fröhlich polarons with high mobility can be created and confined at the surface of SrTiO_3_.

## Discussion

The ultimate collapse of 2D Fröhlich polaron to 3D liquid is puzzling and deserves further examination. Here we interpret it exclusively from the point of view of symmetry. Considering that the EPC is local and linear, in lattice with space inversion symmetry, only phonons with even parity can contribute to the EPC. The linear contribution from polar LO phonons becomes possible only with inducing symmetry breaking. Intrinsic SrTiO_3_ has a centre-symmetric structure, with *d*_*xy*_ and *d*_*xz/yz*_ orbitals degenerate at the bottom of conduction band^41^. As annealed from 800 °C to 1,000 °C, the oxygen vacancies mainly locate at the O1 site (Fig. 1a). These highly restricted 2D defect dipoles break the inversion symmetry along *z* direction, resulting in the level splitting between *d*_*xy*_ and *d*_*xz*_ orbitals as shown in Fig. 5c. The sign change of splitting between 700 °C and 800 °C cases may attribute to the alignment and correlation of dipolar moment. Nevertheless, Franck–Condon line shape fitting yields similar phonon energy and coupling strength (Fig. 4c), indicating the robustness of Fröhlich polaron with broken inversion symmetry. In the case of 1,200 °C, oxygen vacancies expand to 3D and start to occupy O2 sites. These 3D defect dipoles generate relatively even potential along all the three directions. Thus somehow the surface lattices regain its symmetry, deduced by the re-degenerate orbital bands (see Fig. 5c for 1,200 °C case). Consequently, EPC fails, and the many-body effect is dominated by electron–electron interaction, forming 3D liquid. Our scenario suggests that, besides vacuum annealing, other symmetry-breaking alternatives such as applying electric field^42^ or capping layer^6^,^43^ may also initiate the electron–LO phonon coupling and create Fröhlich polarons.

The 2D Fröhlich polaron density falls in the range of which superconductivity in perovskite oxide interfaces is observed^42^,^44^. It has been long proposed that a Bose–Einstein condensation of 2D bipolarons into a superconducting fluid be responsible for the high *T*_c_ in cuprates with multiple LO-phonon branches^45^. The recent discovery of high *T*_c_ in single unit cell thick iron selenide film (1UC FeSe) grown on SrTiO_3_ substrate also suggests the intimate interplay between charge carriers in FeSe and LO-phonon in SrTiO_3_ (refs 6, 46, 47). The transport measurements^48^ first confirm the very high-*T*_c_ superconductivity in single layer FeSe/STO system, in agreement with ref. 6. So if one considers these works together, it would be direct to deduce that this EPC is intrinsic for SrTiO_3_ and be responsible for the enhanced superconductivity in FeSe/STO system. However, even if there are several articles suggesting that the EPC of the SrTiO_3_ may be relevant in the mechanism of the high-*T*_c_ superconductivity, our manuscript is not dealing directly with this issue, in consequence further studies should be carried out to prove or disprove the relation between these two processes.

It is worthy to note that the phonon mode we observed here may not necessarily represent only one LO-branch, but could correspond to the approximation of multiple LO-phonon branches^49^. In this case, the calculated effective coupling constant *α*_3D_ is 2.34(m*)^1/2^. Using the band effective mass (m*) of *d*_*xy*_ orbital in 3D liquid case, we have *α*_3D_∼2.45, in excellent agreement with our experimental value. In this approximation, large bipolarons are found to have an extended stability region at low temperature. Thus our observation of Fröhlich polaron liquid in SrTiO_3_ suggests significant relevance of large (bi)polaron for superconductivity and should stimulate broad interest in exploring polaron behaviour in a wide range of perovskite-related materials, especially high-*T*_c_ cuprates and iron-based superconducting film on SrTiO_3_ substrate.

## Methods

### Sample preparation

SrTiO_3_ (100) single crystals (crystal GmbH and SurfaceNet GmbH) were mounted on resistive Si heaters and cleaned by thermal annealing in vacuum conditions. Each sample was sequentially heated up to particular temperatures, and kept for 20 min, till characteristic LEED patterns are stabilized. At all stages of the cleaning procedure, the pressure was <10^−9^ mbar. All samples presented reproducible and stable phases, with a series of typical reconstructions.

### X-ray Absorption Spectroscopy (XAS)

For the XAS measurements, from the same sample surface secondary electrons were recorded by Scienta energy analyzer, while fluorescence was recorded simultaneously by Bruker detector. XAS spectra simulation was performed by tetrahedral cluster model calculation. The cluster consists of a central Ti atom and its nearest neighbour ligand atoms, and the model calculation includes not only the full multiplets of *3d* electrons but also configuration interaction.

### ARPES

The angle and energy resolutions of the ARPES measurements (Scienta R4000) are set as 0.2^o^ and ∼10 meV. The photon energy-dependent ARPES experiments were performed with photon energy varying from 70 eV to 115 eV. The *kz* was calculated by 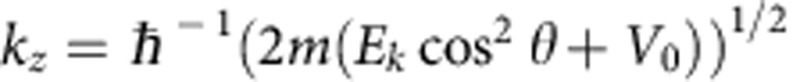. The inner potential *V*_0_=12 eV. Unless otherwise stated, we used photon energy at 100 eV for normal ARPES measurements. The 2D and 3D carrier density can be calculated from the Luttinger area of the FSs. 
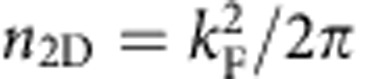
 and 
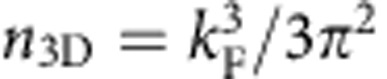
. From the Ti^3+^ ratio, the carrier density can also be calculated by *n*_2D_=ratio × *a*^−2^ and *n*_3D_=ratio × *a*^−3^ with the lattice constant *α*=3.9 Å.

## Additional information

**How to cite this article:** Chen, C. *et al.* Observation of a two-dimensional liquid of Fröhlich polarons at the bare SrTiO_3_ surface. *Nat. Commun.* 6:8585 doi: 10.1038/ncomms9585 (2015).

## Supplementary Material

Supplementary Information Supplementary Figures 1-5
