# Supplementary material for: Observation of a two-dimensional liquid of Fröhlich polarons at the bare SrTiO3 surface
Source: Nat Commun. 2015 Oct 22;6:8585. doi: 10.1038/ncomms9585 (PMC4639792; doi:10.1038/ncomms9585)
Supplement: Supplementary Information — Supplementary Figures 1-5 [file ncomms9585-s1.pdf]

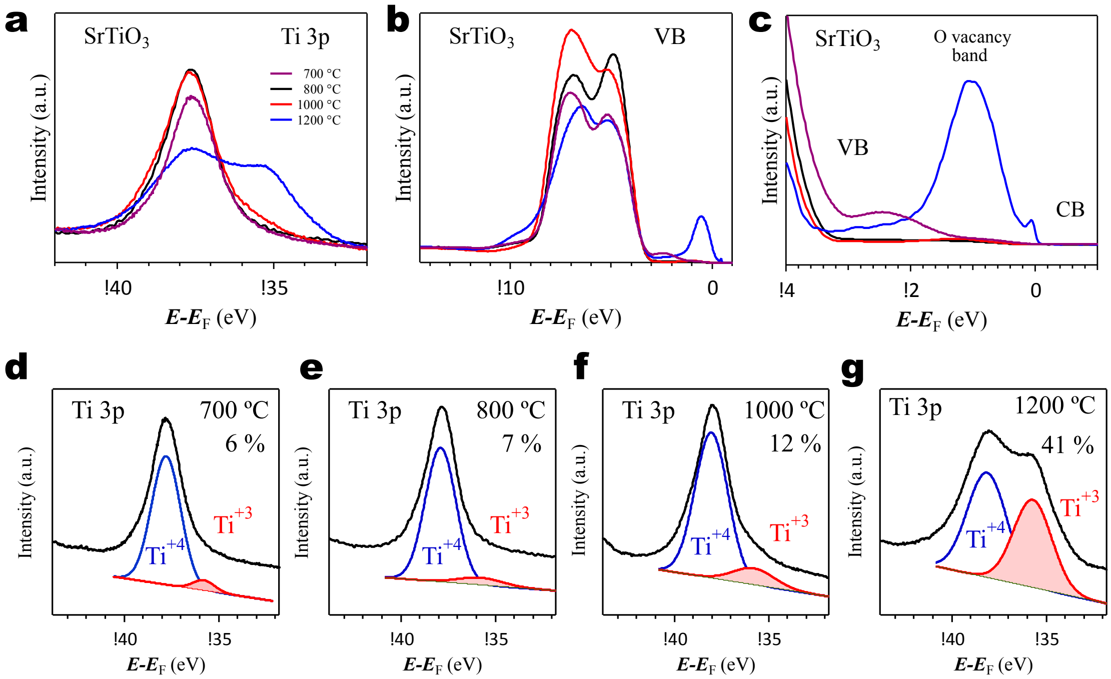

Supplementary Figure 1: Evolution of core levels and valence bands from photoemission spectra. We use photon energy 90 eV to measure the core levels and valence band (angle-integrated) of SrTiO3 surfaces prepared at different temperature. **(a, b, c)** Ti 3p core level, valence band and O vacancy band spectra. All the spectra are normalised to have the same background. **(d, e, f, g)** Evolution of Ti^{3+} ratio with annealing temperature. All the experimental spectra are fitted with two Gaussian peaks and one linear background.

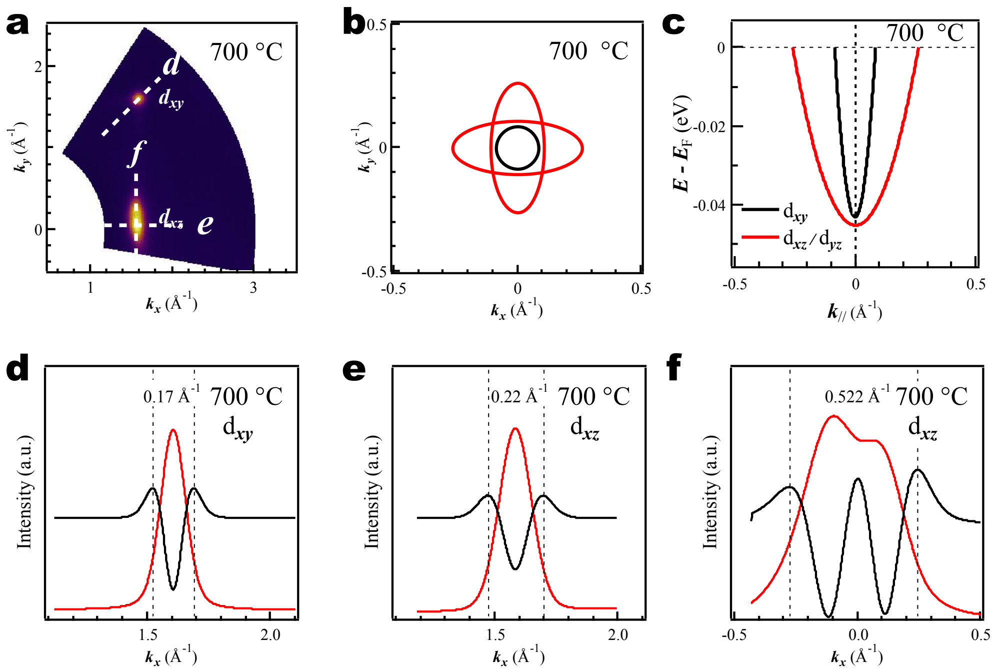

Supplementary Figure 2: MDCs analyses to determine the Fermi surface size of $SrTiO_3$. Exemplary Fermi surface measured for surface prepared at 700 ^oC is shown in **a**. The white dashed line indicates the cuts where MDCs shown at **d, e, f** are from. The red lines in **d, e, f** are original MDCs and black lines are the second derivatives. Black dashed lines indicate the momentum positions for the maximum of the black peaks, defining the Fermi momentum $\pm k_F$ for the corresponding FS. The EDCs fit shown in Fig. 4c in Main text give values of the bottom of band E_b. Assuming a parabolic band shape, the dispersions can be fitted for both orbitals, as shown in **c**. Similarly, assuming an elliptic shape for the d_{xz}/d_{yz} FSs and circular shape for d_{xy} FS, the FSs can be fitted for all the orbitals as shown in **b**. It is worthy to note that the second derivatives show two well-defined peaks, which the left and right one symmetric to the central peak (negative or positive). This offers us an important reference which we can rely on to calculated the size of certain Fermi surfaces which are not completely measured, such as in the case of 1000 ^oC and 1200 ^oC in Supplementary Figure 5.

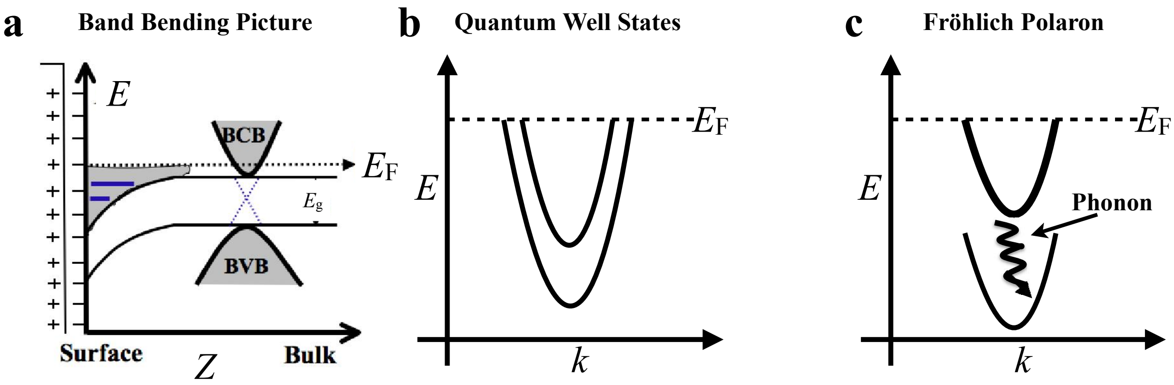

Supplementary Figure 3: **Schematic picture for the formation of Quantum Well States and Fröhlich polaron.** **(a)** The downward potential bending creates a potential well close to the surface. Electrons from the bottom of conduction band can be confined in this potential well. If the potential well is narrow and deep enough, quantum confinement will result in sub-bands as shown in **b**. (**c)** The formation of Fröhlich polaron. The entire conduction band bottom is scattered by one particular phonon mode to higher binding energy, with the original band shape reserved, due to the fact that only q ~ 0 phonons contributed to this coupling.

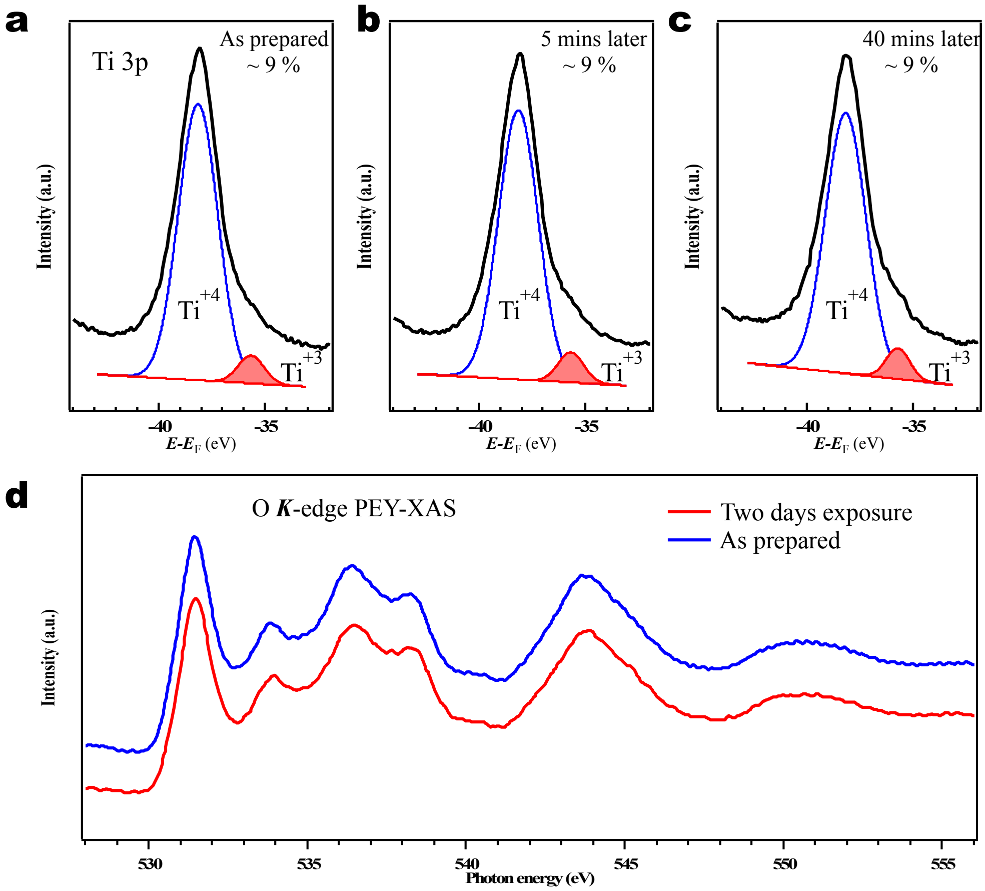

Supplementary Figure 4: **Evolution of $SrTiO_3$ surface electronic structure with irradiation. (a, b, c)** Ti 3p core level spectra taken after different periods of irradiation. **(d)** Oxygen *K*-edge PEY-XAS spectra taken for as-prepared surface and after two-days of irradiation exposure. All the spectra were taken for the same sample with the beam spot at the same position on the sample surface.

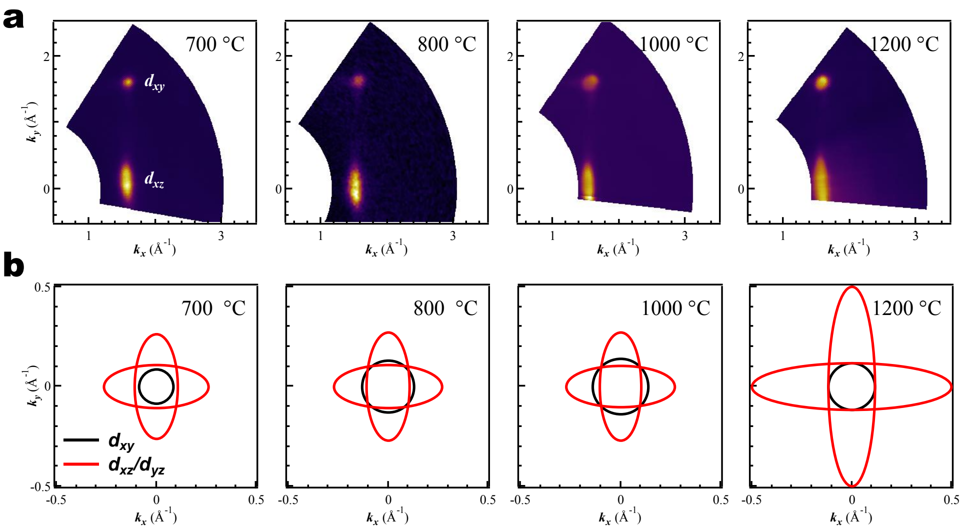

Supplementary Figure 5: **Evolution of Fermi surfaces of SrTiO3 . (a)** Raw data Fermi surfaces measured for surface prepared at different temperature. **(b)** Fitted Fermi surfaces using the method described in Supplementary Figure 2. These are the same FSs shown in main text Figure 5b.
